# The New Genus *Caulinema* Revealed New Insights into the Generic Relationship of the Order Ulotrichales (Ulvophyceae, Chlorophyta)

**DOI:** 10.3390/microorganisms12081604

**Published:** 2024-08-06

**Authors:** Tatyana Darienko, Cecilia Rad-Menéndez, Thomas Pröschold

**Affiliations:** 1 Research Department for Limnology, Leopold-Franzens-University of Innsbruck, A-5310 Mondsee, Austria; tdarien@gwdg.de; 2 Department of Applied Bioinformatics, Institute for Microbiology and Genetics, Georg-August-University of Göttingen, D-37077 Göttingen, Germany; 3 Collection of Algae and Protozoa, Scottish Association for Marine Science, Oban PA37 1QA, UK; cecilia.radmenendez@sams.ac.uk

**Keywords:** *Caulinema*, ITS-2 secondary structure, SSU/ITS phylogeny, *tuf*A phylogeny, *Ulosarcina*

## Abstract

Traditionally, the order Ulotrichales comprised green algae of an unbranched, uniseriate, filamentous morphology. However, since the establishment of ultrastructural features, the circumscription of this order has dramatically changed. Some genera and species have been excluded from this order and others with different morphologies (sarcinoid, branched filaments or even parenchymatous taxa) have been included. Phylogenetic analyses have confirmed the monophyly of this order, but its differentiation from the Ulvales and Acrosiphoniales remains difficult because of the lack of synapomorphies at every level (morphology, molecular signatures). To demonstrate the difficulties of placement into genera and orders, we investigated two sarcinoid taxa with the absence of zoospore formation. SSU and ITS rDNA tree topology and the ITS-2/CBC approach revealed that both strains SAG 2661 and CCAP 312/1 belong to *Ulosarcina terrestrica* and the newly erected genus *Caulinema*, respectively. The species conception using this approach was evaluated by sequencing the plastid-coding gene *tuf*A, a commonly used barcode marker for green algae. All three molecular markers resulted in similar topologies at the generic and species levels, which is consistent with the ITS-2/CBC approach and *tuf*A for barcoding. The reevaluation of the ultrastructural features revealed that the presence of organic scales on the surfaces of motile cells is characteristic for the order Ulotrichales and can be used for separation from the closely related orders. As a consequence of our study, we propose the new genus *Caulinema* for strain CCAP 312/1.

## 1. Introduction

Mattox and Stewart [1] established the green algal class Ulvophyceae based on the ultrastructure of flagellated cells (counterclockwise orientation of the basal body apparatus). Originally, they recognized two orders, Ulvales Blackman & Tansley and Ulotrichales Borzi. Since the descriptions, both orders have undergone drastic changes in their circumscriptions and species and generic compositions. For example, most scientists before the molecular era summarized all the unbranched uninucleate filaments with or without the formation of rhizoids in the order Ulotrichales. The history of this order is described in detail in Lokhorst [2]. In contrast, considering the results of ultrastructural investigations, sarcinoid to parenchymatous taxa as well as branched filaments were included [1,3]. The subdivision of green algae based on the morphology of their vegetative cells/thalli was already questioned before the ultrastructure investigations and phylogenetic analyses were undertaken. Kornmann [4,5,6] studied the life cycles of several marine green algae and found the presence of a “*Codiolum*” stage as the unicellular sporophyte in many of these taxa. He concluded that all these green algae are closely related despite their differences in gametophyte morphology. To differentiate these species and genera from the traditional Ulotrichales, he proposed the name Codiolales for this group [4]. This order was not widely accepted because the nomenclature of higher ranks should be based on a representative genus belonging to this order. The genus *Codiolum* A.Braun with its 15 described species is only the unicellular sporophyte of several marine green algae. Kornmann’s conception for the classification of green algae was included in the circumscription of the order Ulotrichales by O’Kelly and Floyd [3]. In addition, they demonstrated that all the investigated Ulotrichales have tiny organic scales of the gametes/zoospores in contrast to those belonging to the Ulvales. Only the so-called *Acrosiphonia* group consisting of the genera *Acrosiphonia* J.Agardh, *Spongomorpha* Kützing, and *Urospora* Areschoug does not produce organic scales on flagellated cells. This group has been considered a separate order by Jónsson [7]; however, this order is not widely accepted despite its monophyly among the Ulvophyceae based on phylogenetic analyses [8]. Other genera, *Chlorocystis* L.Reinhard, and *Halochlorococcum* P.J.L. Dangeard ex Guiry, the *Chlorocystis* group, were also considered to be members of the Ulotrichales by O’Kelly and Floyd [3], belonging to the separate order Chlorocystidales [9,10].

Phylogenetic analyses of several molecular markers (SSU, ITS, *tuf*A) as well as plastomes and transcriptomes revealed the monophyly of the Ulotrichales *sensu* O’Kelly and Floyd with emendations [11,12,13]. At present, the following genera belong to this order: *Sarcinofilum* Darienko & Pröschold; *Tupiella* Darienko & Pröschold; *Chamaetrichon* Tupa; *Vischerioclonium* Darienko & Pröschold; *Rhexinema* Geitler; *Planophila* Gerneck; *Kraftionema* Wetherbee & Verbruggen; *Ulosarcina* Gontcharov et al.; *Eugomontia* Kornmann; *Gomontia* Bornet & Flahault; *Collinsiella* Setchell & Gardner; *Monostroma* Thuret; *Gayralia* Vinogradova; and *Ulothrix* Kützing. The last three genera need to be revised because no cultures and sequences are available from the type species.

During the screening of ulvophycean cultures available in public culture collections and own isolates, we found two sarcinoid taxa with the tendency to form filaments, which could not be identified with the described genera based solely on morphology. The strains SAG 2661 and CCAP 312/1 showed morphological similarities to *Ulosarcina* and *Rhexinema*, respectively, but differed from the ecology of both these genera. The aim of this study was to find out the phylogenetic positions of both taxa and present the molecular synapomorphies of the genera belonging to the Ulotrichales.

## 2. Materials and Methods

### 2.1. Cultures and Light Microscopy

The following strains were investigated:

- The strain CCAP 312/1 was isolated from sand grains in Kames Bay, Millport, Isle of Cumbrae, Scotland, UK (55°45′18″ N, −4°54′56″ E), by Michael Droop in 1958. This marine strain was cultivated in seawater medium (SWES, medium 5 in Schlösser [14]) at 20 °C at a light intensity of 20 µE/m^2^s and under a light:dark cycle of 14:10 h;
- The strain SAG 2661 was isolated from a sample collected from the Seeache by Unterach, Austria (47°48′8″ N, 13°27′1″ E), in 2021. This freshwater strain was grown in Bold’s basal medium (3N-BBM+V, medium 26a in Schlösser [15]) under the same conditions.

To investigate the phenotypic plasticity, both strains were cultivated in different media under the same culture conditions: modified artificial seawater medium (MASM, medium 25 in Schlösser [15]); added 30 mL soil extract per liter; brackish medium (1/2SWES, medium 6 in Schlösser [14]); and basal medium (ES, medium 1 in Schlösser [14]).

The four-week-old cultures were investigated near the end of the light period with an Olympus BX-60 light microscope (Olympus, Tokyo, Japan), which was equipped with a Prog Res C14 plus camera and the Prog Res Capture Pro imaging system (version 2.9.0.1), both from Jenoptik, Jena, Germany, for the documentation of the micrographs.

### 2.2. DNA Extraction, PCR, Sequencing, and Phylogenetic Analyses

The DNA extractions of both strains were performed as described in Darienko et al. [16]. The genomic DNA was extracted using the DNeasy Plant Mini Kit (Qiagen, Hilden, Germany) following the instructions that were provided by the manufacturer. The following PCR amplifications were conducted: (i) The SSU and ITS rDNA sequences were amplified in two steps using the primer combinations EAF3/U1400R and U920F/ITS055R. The primer sequences of EAF3 and ITS055R were published by Marin et al. [17], and the other two primers were newly designed specifically for ulvophytes (U920F: 5′-CAAGGCT-GAAACTTAAAGGAATTG-3′; U1400R: 5′-CAATCGGTAGGAGCGACGGGCGGTGTGTAC-3′). The amplifications were conducted by the following cycle: 5 min initial denaturation at 96 °C followed by 30 cycles each comprising 1 min denaturation at 96 °C, 2 min primer annealing at 55 °C, 3 min elongation at 68 °C, and lastly, 10 min final elongation at 68 °C. (ii) The proposed barcode marker *tuf*A was amplified using the primer combination *tuf*GF4/*tuf*AR [18,19]. For comparison, the *tuf*A of the strains studied by Darienko and Pröschold [11], which belong to the order Ulotrichales, was sequenced. Unfortunately, the PCR amplification was not successful for all of these strains. Therefore, we designed two new primers called U*tuf*AF1 (5′-GGNCAYGCNGAYTAYGTAAAAA-AYATG-3′) and U*tuf*AF2 (5′-GGNGCNGCNCAAATGGAYGGWGC-3′) and combined them with *tuf*AR. The *tuf*A amplification was conducted by the following cycle: 4 min initial denaturation at 94 °C followed by 38 cycles each comprising 1 min denaturation at 94 °C, 30 s primer annealing at 45 °C, 1 min elongation at 72 °C, and lastly, 7 min final elongation at 68 °C. The PCR products were purified using the QIAquick PCR Purification Kit (Qiagen, Hilden, Germany) following the instructions provided by the manufacturer. The sequencing of the purified PCR products was performed with the following primers (N82F, E528F, U920F, 536R, U1400R, BR, GF, and GR; primer sequences published in [13]) for the SSU and ITS sequences. The *tuf*A products were sequenced using the primers U*tuf*AF2 and *tuf*AR.

The SSU and ITS rDNA sequences were manually aligned according to their secondary structures, as described in Darienko and Pröschold [11]. For aligning the SSU, the structure of SAG 38.86 *Ulothrix zonata* (see Figure S1 in [11]) was used as a template. The sequences of *tuf*A were aligned manually after checking them at the amino acid level. For phylogenetic analyses, all sequences were included in several datasets: (i) The SSU rDNA sequences were included in a large dataset of 64 taxa of representatives belonging to the Ulvophyceae *s.str.* with 1780 unambiguously aligned positions to find out to which order both new taxa belong. (ii) To obtain a higher resolution among the Ulotrichales, a concatenated dataset of SSU and ITS consisting of 36 taxa with 2354 unambiguously aligned positions was created. (iii) For the comparison of *tuf*A and ITS rDNA, both were separately concatenated with SSU into two datasets (SSU + ITS: 33 taxa with 2354 bases; SSU + *tuf*A: 33 taxa with 2631 bases). (iv) To decide whether ITS-2 (commonly used as a marker at the species level) and *tuf*A (commonly used as a barcode marker) produce similar results in phylogenetic analyses, three datasets of the 33 taxa were created (ITS-2: 242 bp; *tuf*A using all 3 codon bases: 861 bp; *tuf*A using the first 2 codon bases: 574 bp). (v) Finally, the three genes were concatenated for a comparison with the results of the analyses of the separated datasets (33 taxa with 3215 bases).

All datasets were analyzed with the program PAUP* version 4.0a (build 169; [20]). The best fitted evolutionary model was calculated with the automated model selection tool implemented in PAUP. The settings of the best models are given in the figure legends. EMBL/GenBank accession numbers of published sequences and strain designations are provided in these figures. Phylogenetic trees were inferred using distance, parsimony, and maximum likelihood criteria using PAUP [20], and the robustness of the tree topologies was proven by different Bayesian and bootstrap analyses (1000 replicates). In addition, the programs RAxML version 8.2.12 [21], MrBayes version 3.2.7a [22], and PHASE package 2.0 [23,24,25,26,27] were used.

The secondary structures of the ITS-2 sequences were folded using mfold [28] of the UNAFold platform (http://www.unafold.org/RNA_form.php, accessed on 28 July 2024) according to the approach introduced by Darienko and Pröschold [11] for non-marine ulvophytes. The visualization of the structures was conducted with the program VARNA version 3.93 [29]. The conserved region of ITS-2 was used to discover compensatory base changes (CBCs) for species delimitation (ITS-2/CBC approach *sensu* Darienko and Pröschold [11]).

## 3. Results

### 3.1. Morphology and Phenotypic Plasticity

**Figure 1:**
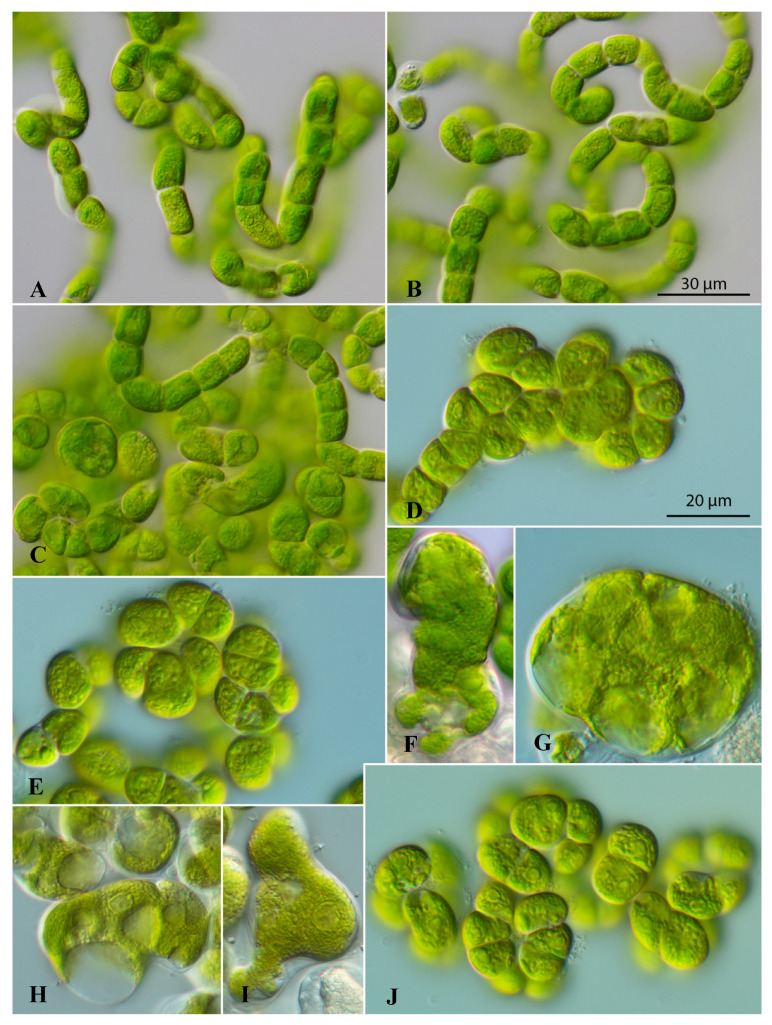
Morphology and phenotypic plasticity of *Caulinema droopii* (CCAP 312/1) grown on SWES medium. (**A**–**C**) Different filamentous stages; (**D**,**E**,**J**) different sarcinoid stages; (**F**–**I**) different *Codiolum*-like stages. Scale bar in (**A**–**C**) = 30 µm; scale bar in (**D**–**J**) = 20 µm.

The strain CCAP 312/1 formed two morphological stages: filamentous and sarcinoid with intermediate stages (Figure 1). The filaments were unbranched and very curly, resulting in node-like structures. Sometimes they disintegrated into sarcinoid packages consisting of two–eight cells. The vegetative cells were mostly cylindrical to squared, and the end cells were rounded. The chloroplasts were parietal containing a single pyrenoid. The reproduction occurred by vegetative cell division and no zoospore formation was observed. Interestingly, a *Codiolum*-like stage could be observed in old cultures, which indicated that this strain belongs to the Ulvophyceae. Comparing the morphology of this strain with already described genera, it showed some similarity with *Rhexinema* (see [11]).

**Figure 2:**
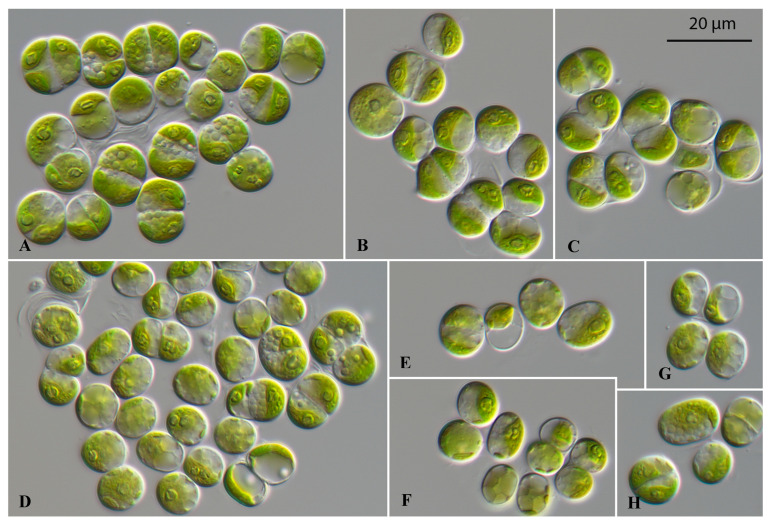
Morphology and phenotypic plasticity of *Ulosarcina terrestrica* (SAG 2661) grown on 3N-BBM + V medium. (**A**–**H**) Different sarcinoid and coccoid stages. Scale bar = 20 µm.

The strain SAG 2661 (Figure 2) is similar in morphology to *Ulosarcina terrestrica*, described by Gontcharov et al. [30]. The vegetative cells were broadly ellipsoidal, mostly solitary, and formed packages in the surrounding mucilage. The parietal chloroplasts contained a single pyrenoid. Old cells were often vacuolized. In contrast to the original description of *Ulosarcina terrestrica*, we could not observe any biflagellated zoospores. The asexual reproduction occurred by vegetative cell division.

To test the phenotypic plasticity of both strains, we tried to cultivate them in different freshwater (3N-BBM+V and ES), brackish (1/2SWES), and marine (MASM and SWES) media. The strain CCAP 312/1 grew only on the medium containing natural seawater (SWES). In contrast, SAG 2661 was only able to grow on freshwater media without any changes in morphology, but it also did not show any growth on brackish and marine media.

### 3.2. Phylogenetic Position of Both Investigated Strains

**Figure 3:**
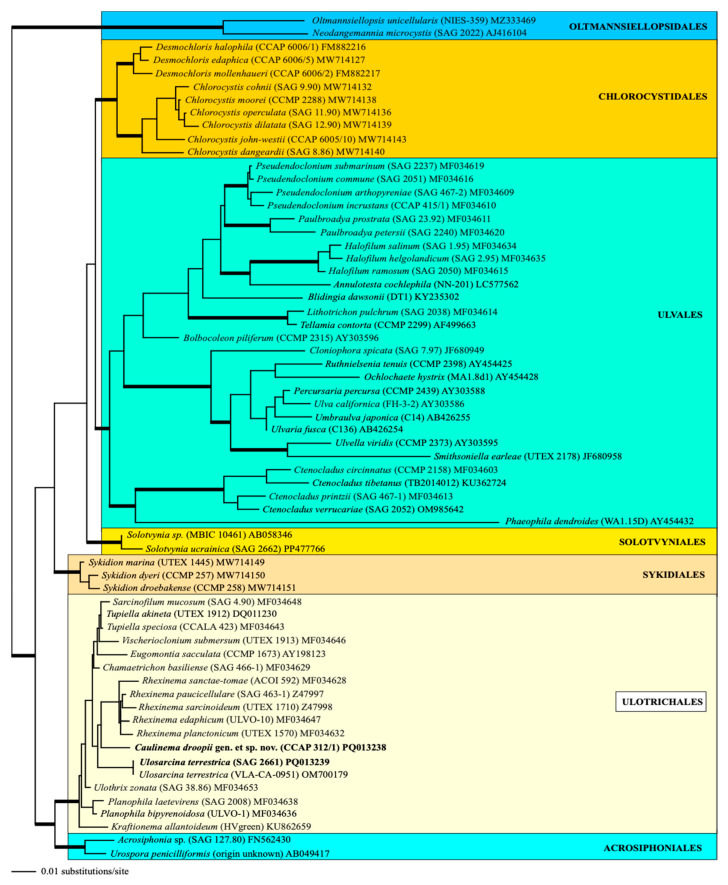
Molecular phylogeny of the Ulvophyceae s. str. based on SSU rDNA sequence comparisons. The phylogenetic tree shown was inferred using the maximum likelihood method based on a dataset of 1777 aligned positions of 64 taxa using PAUP 4.0a build169. For the analysis, the GTR + I + G (base frequencies: A 0.23431; C 0.22549; G 0.28486; U 0.25534; rate matrix A-C 1.2900; A-G 2.3030; A-U 1.3336; C-G 0.6993; C-U 4.2187; G-U 1.0000) with the proportion of invariable sites (I = 0.5543) and the gamma shape parameter (G = 0.4520) was chosen, which was calculated as the best model by the automated model selection tool implemented in PAUP. The branches in bold are highly supported in all analyses (Bayesian values >0.95 calculated with PHASE and MrBayes; bootstrap values >70% calculated with PAUP using maximum likelihood, neighbor joining, maximum parsimony, and RAxML using maximum likelihood). The sister group Oltmannsiellopsidales was chosen as an outgroup. The clade designations follow the currently accepted order classification of the Ulvophyceae [10]. The newly sequenced strains are highlighted in bold.

**Figure 4:**
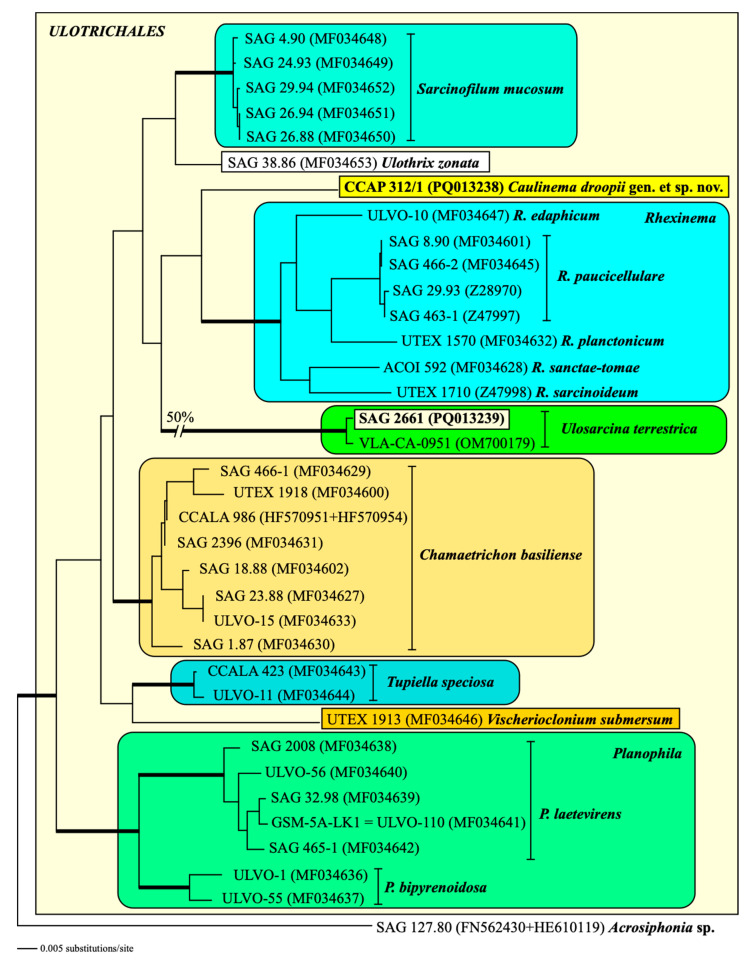
Molecular phylogeny of the Ulotrichales based on SSU and ITS rDNA sequence comparisons. The phylogenetic tree shown was inferred using the maximum likelihood method based on the datasets (2354 aligned positions of 36 taxa) using PAUP 4.0a build169. For the analyses, the best model was calculated by the automated model selection tool implemented in PAUP. The setting of the best model was given as follows: SYM + I + G (base frequencies: equal; rate matrix A-C 1.2047; A-G 2.7302; A-U 1.4807; C-G 0.6519; C-U 5.6806; G-U 1.0000) with the proportion of invariable sites (I = 0.7257) and gamma shape parameter (G = 0.4521). The branches in bold are highly supported in all analyses (Bayesian values >0.95 calculated with PHASE and MrBayes; bootstrap values >70% calculated with PAUP using maximum likelihood, neighbor joining, maximum parsimony, and RAxML using maximum likelihood). The newly sequenced strains are highlighted in bold.

Phylogenetic analyses of the SSU rDNA sequences clearly revealed that both strains belong to the order Ulotrichales of the Ulvophyceae (Figure 3). The SSU rDNA of strain SAG 2661 is completely identical in sequence to the authentic strain of *Ulosarcina terrestrica*, which was originally described by Gontcharov et al. [30]. In contrast, the strain CCAP 312/1 represents an own lineage within the Ulotrichales, closely related to the genus *Rhexinema*. However, the resolution is weak, caused by the low genetic variability in the SSU rDNA within this order (<3% variability in uncorrected p-distances). The bootstrap and Bayesian support of all orders belonging to the Ulvophyceae is very high (>95% in all bootstrap analyses and >0.95 in all Bayesian analyses). For a higher resolution within the Ulotrichales, we used the concatenated dataset of SSU and ITS rDNA sequences. Compared with the SSU dataset used for Figure 3, this dataset is slightly reduced because of the lack of ITS rDNA sequences from *Eugomontia* and *Kraftionema*. Among the Ulotrichales, the analyses of this dataset showed nine lineages representing genera with high bootstrap and Bayesian support. Both newly sequenced strains, SAG 2661 and CCAP 312/1, represented *Ulosarcina* and a new genus, *Caulinema* (described below), respectively (Figure 4).

### 3.3. ITS-2/CBC Approach of the Ulotrichales for Species Delimitation

**Figure 5:**
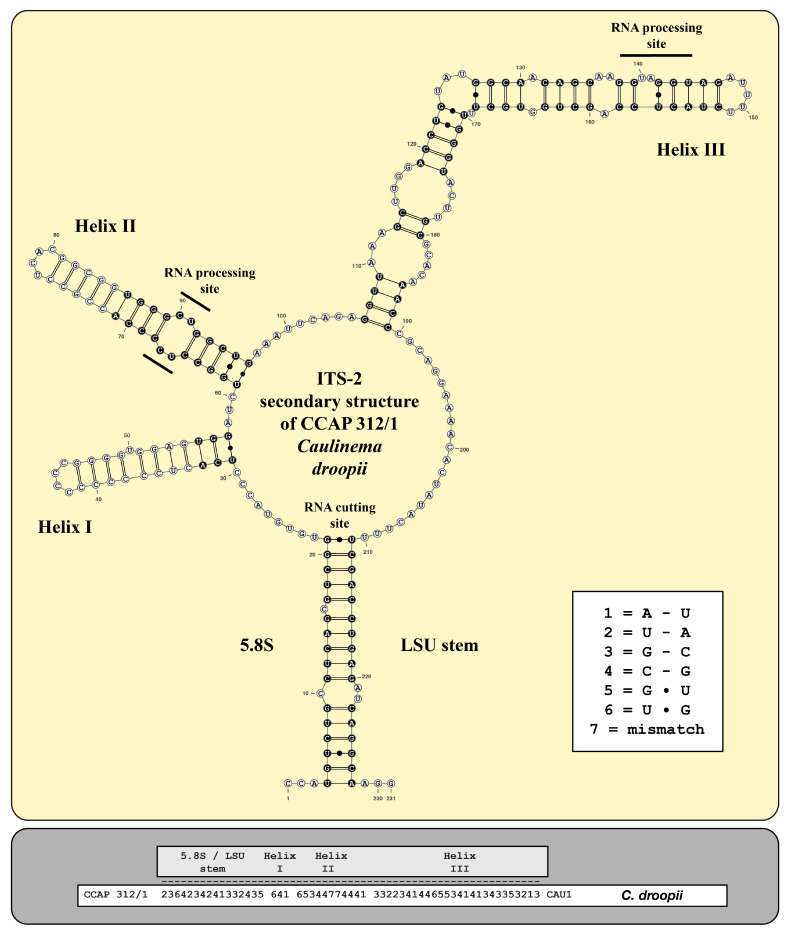
ITS-2 secondary structure and barcode of *Caulinema droopii*. The extraction of the conserved region (highlighted in black circles with white bases) and the translation into a number code for its usage as a barcode.

**Figure 6:**
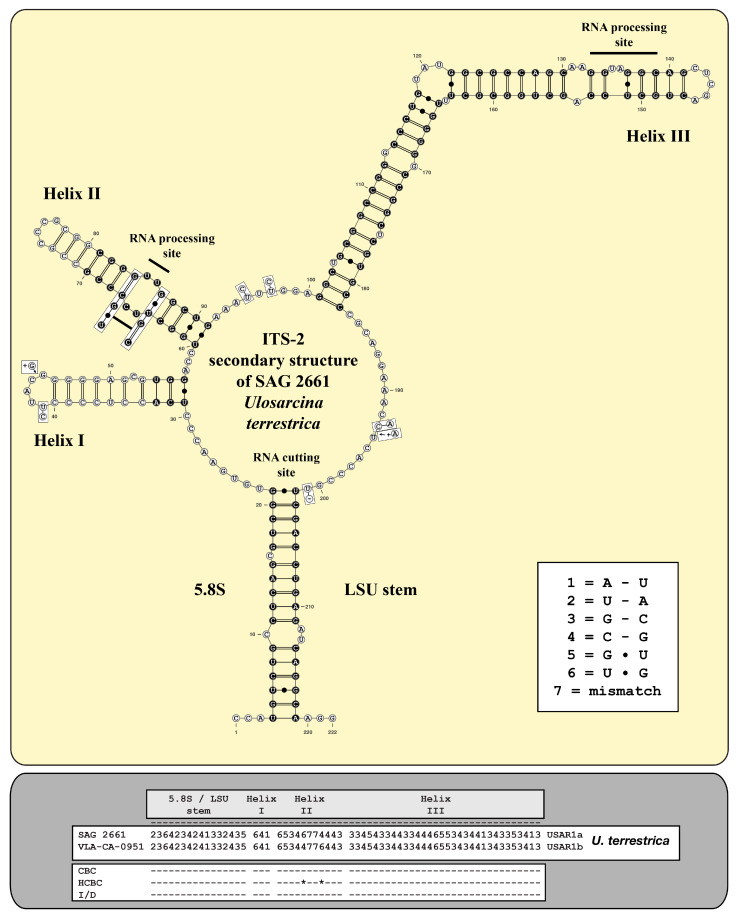
ITS-2 secondary structure and barcode of *Ulosarcina terrestrica*. The extraction of the conserved region (highlighted in black circles with white bases) and the translation into a number code for its usage as a barcode. The changes in SAG 2661 and the authentic strain of *Ulosarcina terrestrica* are highlighted in the white boxes.

**Figure 7:**
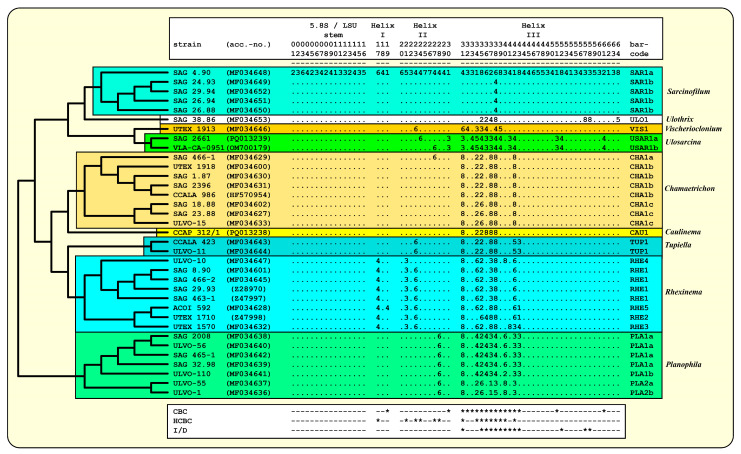
Comparison of the conserved region of ITS-2 among the Ulotrichales. The extraction of this region and the translation into a number code for its usage as a barcode. Number code for each base pair: 1 = A-U; 2 = U-A; 3 = G-C; 4 = C-G; 5 = G•U; 6 = U•G; 7 = mismatch; 8 = deletion, single or unpaired bases. The presence of CBCs, HCBCs, and deletion, single or unpaired bases (I/D) is marked with an asterisk (*).

As already demonstrated for different lineages of the Ulvophyceae, Darienko and Pröschold [11] and Darienko et al. [10] used the ITS-2/CBC approach for species delimitation. For comparison, the ITS-2 secondary structures of the newly investigated strains were folded using the approach described by Darienko and Pröschold [11]. The ITS-2 secondary structures of the strains CCAP 312/1 and SAG 2661 are very conserved, as is typical for Ulvophyceae, and have three helices (Helix I-III *sensu* Mai and Coleman [31]); Helix IV is missing (Figure 5 and Figure 6). The conserved regions of ITS-2 highlighted in black circles in Figure 5 and Figure 6 were used as the barcodes, which were then translated into number codes. These numeric barcodes were then compared with all the ulotrichalean strains presented in Figure 4. This number alignment was analyzed using the neighbor-joining method implemented in PAUP to visualize the data as a tree. The resulting tree topology is presented in Figure 7, together with the number barcode for each strain. The nine genera and their species could be clearly distinguished by unique barcodes. Only the relationship among the genera differed from the tree presented in Figure 4. Among the Ulotrichales, 17 compensatory base changes (CBCs) and 15 HCBCs (one-sided CBCs) could be recognized (all marked with an asterisk in Figure 7). The genera with more than one species also showed CBCs between their species (*Rhexinema—*five species: seven CBCs, two HCBCs; *Planophila—*two species: four CBCs, four HCBCs). The ITS-2 barcode of CCAP 312/1 showed similarities to those of *Chamaetrichon* strains but differed in four positions from them. The ITS-2 of strain SAG 2661 showed few differences to the authentic strain of *Ulosarcina terrestrica*, as demonstrated in the white boxes in Figure 6. Comparing the ITS-2 barcodes of both strains, two HCBCs in Helix II could be discovered. Interestingly, both strains have identical SSU, ITS-1, and 5.8S rDNA sequences but differ in ITS-2, which is unusual. In contrast, different strains of the same species mostly showed genetic variability in ITS-1 but were similar in ITS-2, which can be seen for *Chamaetrichon basiliense* or *Rhexinema paucicellulare*.

### 3.4. The Usage of the Chloroplast-Encoded Barcode Marker tufA among the Ulotrichales

**Figure 8:**
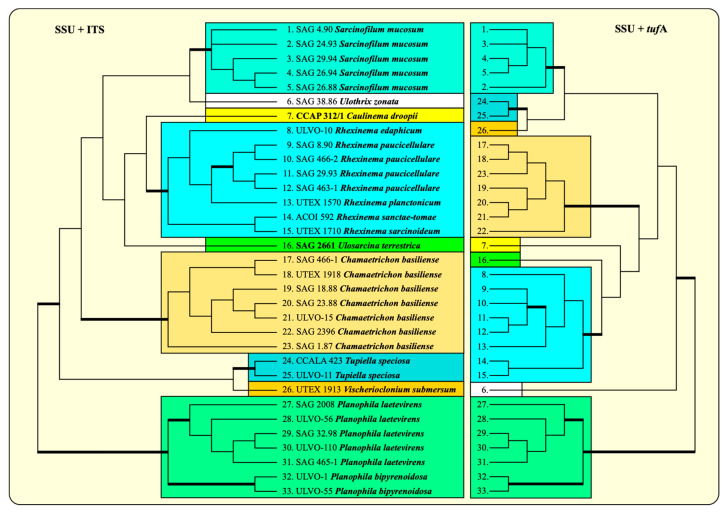
Comparison of the ITS rDNA and *tuf*A of the Ulotrichales (concatenated with SSU rDNA sequences). The phylogenetic trees shown were inferred using the maximum likelihood method based on the two datasets (SSU + ITS: 33 taxa with 2354 bases; SSU + *tuf*A: 33 taxa with 2631 bases) using PAUP 4.0a build169. For the analyses, the best model was calculated by the automated model selection tool implemented in PAUP. The settings of the best model were given as follows: for SSU + ITS: SYM + I + G (base frequencies: equal; rate matrix A-C 1.2342; A-G 2.7833; A-U 1.4845; C-G 0.7471; C-U 6.1015; G-U 1.0000) with the proportion of invariable sites (I = 0.7492) and gamma shape parameter (G = 0.4714) and for SSU + *tuf*A: GTR + I + G (base frequencies: A 0.2839; C 0.1779; G 0.2457; U 0.2925; rate matrix A-C 1.7555; A-G 3.2543; A-U 1.7332; C-G 1.6119; C-U 5.0818; G-U 1.0000) with the proportion of invariable sites (I = 0.7473) and gamma shape parameter (G = 0.6406). The branches in bold are highly supported in all analyses (Bayesian values >0.95 calculated with PHASE and MrBayes; bootstrap values >70% calculated with PAUP using maximum likelihood, neighbor joining, maximum parsimony, and RAxML using maximum likelihood).

The elongation factor Tu (*tuf*A) encoded in the chloroplast has been proposed as a barcode marker for green algae [19]. Therefore, we sequenced this gene of all the available strains presented in Figure 4, with the exception of the strains CCALA 986 *Chamaetrichon basiliense* and VLA-CA-0951 *Ulosarcina terrestrica* (both not available at present). To find out whether the *tuf*A has the same discrimination power as ITS, we combined the *tuf*A sequences with the SSU into one dataset and compared the phylogeny of this dataset with the SSU + ITS. The phylogenetic analyses of both datasets revealed similar tree topologies. All genera and species were clearly recognizable, and only the relationship among the genera differed (Figure 8).

**Figure 9:**
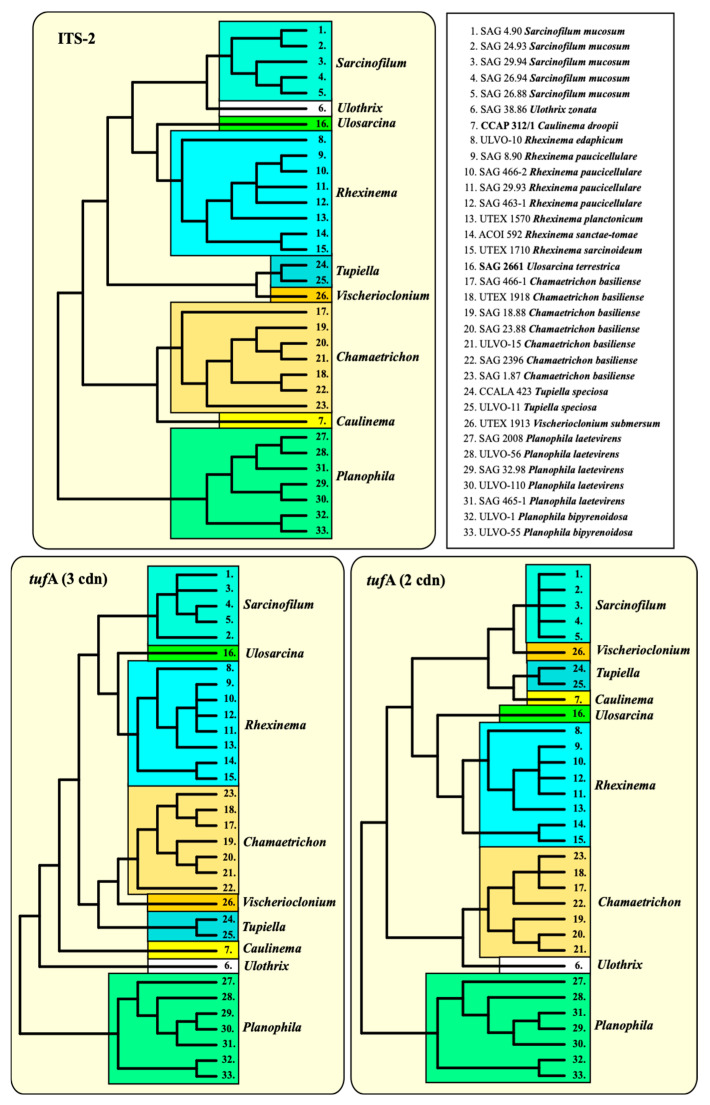
Comparison of the ITS-2 rDNA and *tuf*A (all three and only the first codon bases) among the Ulotrichales (33 taxa).

**Figure 10:**
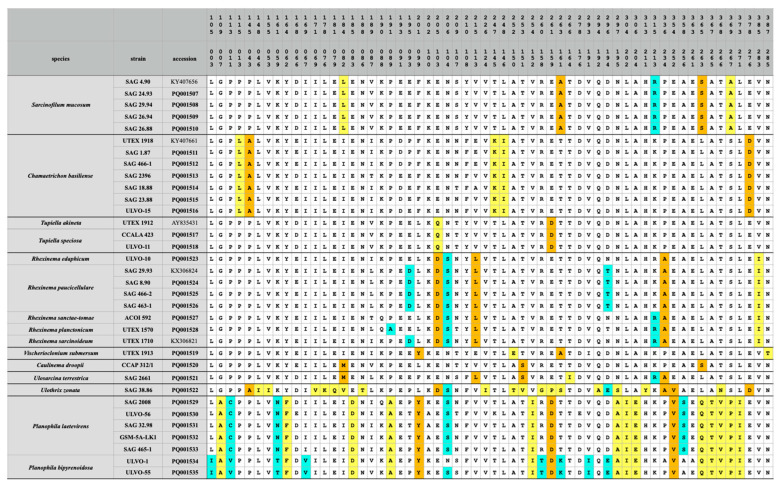
Variable positions in the *tuf*A amino acid pattern among the Ulotrichales. The abbreviations of amino acids followed the IUPAC nomenclature. The unique positions of the genera and species are highlighted in yellow and green, respectively. Asymmetric positions (not unique for only one genus) are marked in orange. The given numbers in the top represented the amino acid position in the *tuf*A gene, the numbers below were the positions in the alignment. The accession numbers in bold were newly sequenced in this study.

To figure out whether ITS-2 and *tuf*A can also be used without the combination with SSU, three datasets (ITS-2, *tuf*A with all codon bases and *tuf*A with only the first two codon bases) were analyzed, as described in the Material and Methods. The phylogenetic analyses clearly confirmed the monophyly of each genus, as already demonstrated in all the previous figures. Only the relationship among the genera remained unresolved (Figure 9). For finding molecular signatures of *tuf*A, which could be used as diagnostic features for genera and species, the *tuf*A sequences were transferred into amino acids. The variable positions of the alignment consisting of 287 amino acids were discovered and are summarized in Figure 10. A total of 60 amino acids varied among the Ulotrichales. Most of them were diagnostic at the generic and species levels. A total of 35 of them (highlighted in yellow in Figure 10) were unique at the generic level, and 13 (highlighted in green) were unique at the species level. In addition, 12 positions (highlighted in orange) could be used as significant at the generic level if used in combination with the complete pattern.

## 4. Discussion

### 4.1. The Molecular Phylogeny and Systematics of the Order Ulotrichales

As already mentioned above, the circumscription of the order Ulotrichales has a long history [2,3]. The traditional description (unbranched, uninucleated filaments) using exclusively gametophyte features has been dramatically revised by the establishment of ultrastructural investigations in the systematics of these green algae. The basal body orientation of the motile cells and the type of mitosis were used for the characterization of the Ulvophyceae [1]. In addition, O’Kelly and Floyd [2] included life cycle features in the circumscription of the Ulotrichales, which was originally proposed by Kornmann [4,5,6]. Sluiman [32] summarized the ultrastructural features of the Ulvophyceae as follows: (i) a counterclockwise orientation of motile cells, and (ii) closed mitosis during cell division (Type V *sensu* van den Hoek et al. [33,34]). However, neither of these features differentiate the ulvophytes at the order level. Interestingly, the presence of organic scales as described by Floyd and O’Kelly [35] was not used for the differentiation of the Ulotrichales from the Ulvales. According to Sluiman [32] and the references therein, organic scales on the surfaces of motile cells have been reported for the following taxa: *Vischerioclonium submersum* (assigned as *Pseudendoclonium basiliense*; [36]); *Tupiella akineta* (assigned as *Pseudendoclonium akinetum*; [35]); *Sarcinofilum mucosa* (assigned as *Trichosarcina polymorpha*; [35,36]); *Monostroma grevillei* [37,38]; *M. bullosum* [39]; *Gayralia oxysperma* (assigned as *Monostroma*; [39,40]); *Ulothrix zonata* [41,42]; *U. mucosa* [43]; and *Eugomontia sacculata* [35]. Nakayama and Inouye [44] also found square scales on the surfaces of gametes by *Collinsiella cava* and concluded that this species is closely related to *Monostroma* among the Ulotrichales. Similar square scales were also reported for another strain of *Chamaetrichon basiliense* and *Rhexinema sarcinoidea* (called *Chamaetrichon capsulatum* and *Protoderma sarcinoidea*, respectively) [45]. Friedl [46] also observed scales on the surfaces of zoospores by the authentic strain of *Rhexinema paucicellulare* (assigned as *Pleurastrum*).

Comparing all these results with the known phylogenetic analyses of the SSU, ITS, *tuf*A, and *rbc*L sequences, all these taxa belong to the Ulotrichales [11,35,46,47,48,49,50]. In addition, Friedl and O’Kelly [51] and Darienko and Pröschold [11] demonstrated that the two species of *Planophila* (*P. laetevirens* and *P. bipyrenoidosa*) also belong to the Ulotrichales. Unfortunately, the ultrastructure of the quadriflagellated zoospores has not been investigated yet. Wetherbee and Verbruggen [52] and Gontcharov et al. [30] described the genera *Kraftionema* and *Ulosarcina*, respectively. Both were also members of the Ulotrichales, as confirmed in our study (see Figure 3). Whereas the authentic strain of the genus *Ulosarcina* produced biflagellated zoospores according to Gontcharov et al. [30], we were not able to observe flagellated cells by SAG 2661. Unfortunately, the zoospores of the authentic strain of *Ulosarcina terrestrica* has not yet been investigated by TEM. Zoospore formation was absent for the genus *Kraftionema* [52] and the newly investigated strain CCAP 312/1, described as the new genus *Caulinema* below. However, the phylogenetic analyses clearly revealed that both represent own lineages within the Ulotrichales. The ITS-2 barcodes and the *tuf*A signature clearly supported the erection of the new genus *Caulinema* for the strain CCAP 312/1 (Figure 3, Figure 4, Figure 5, Figure 6, Figure 7, Figure 8, Figure 9 and Figure 10). Summarizing, at present, the order Ulotrichales consists of taxa with different morphological features, from unicellular/sarcinoid (*Planophila*, *Ulosarcina*, *Gomontia, Caulinema*) and unbranched (*Kraftinema*, *Ulothrix*, *Eugomontia*) and branched (*Chamaetrichon*, *Tupiella*, *Vischerioclonium*, *Rhexinema, Caulinema*) to parenchymatous (*Monostroma*, *Gayralia*, *Collinsiella*) taxa. The last group as well as the species of the genus *Ulothrix* need further investigations because no or very little molecular data, especially of the type species, of these genera are available.

The investigated genera were well characterized by the morphology and molecular phylogeny of the SSU, ITS, and *tuf*A sequences, as well as by the ITS-2/CBC approach and signatures of *tuf*A at the amino acid level. However, as also demonstrated, the relationship among the genera remained unresolved. Therefore, the subdivision of the Ulotrichales into families, as proposed by Skaloud et al. [53], is not practical, especially because molecular data of the type species of some genera (*Ulothrix*, *Monostroma*) are not available and can only be used as preliminary guidelines for identification. Preliminary studies of the genera *Monostroma* and *Ulothrix* revealed that both genera are polyphyletic and need taxonomic revisions [54,55]. At present, if it is necessary to create ranks below the order level, each genus represents its own family.

**Figure 11:**
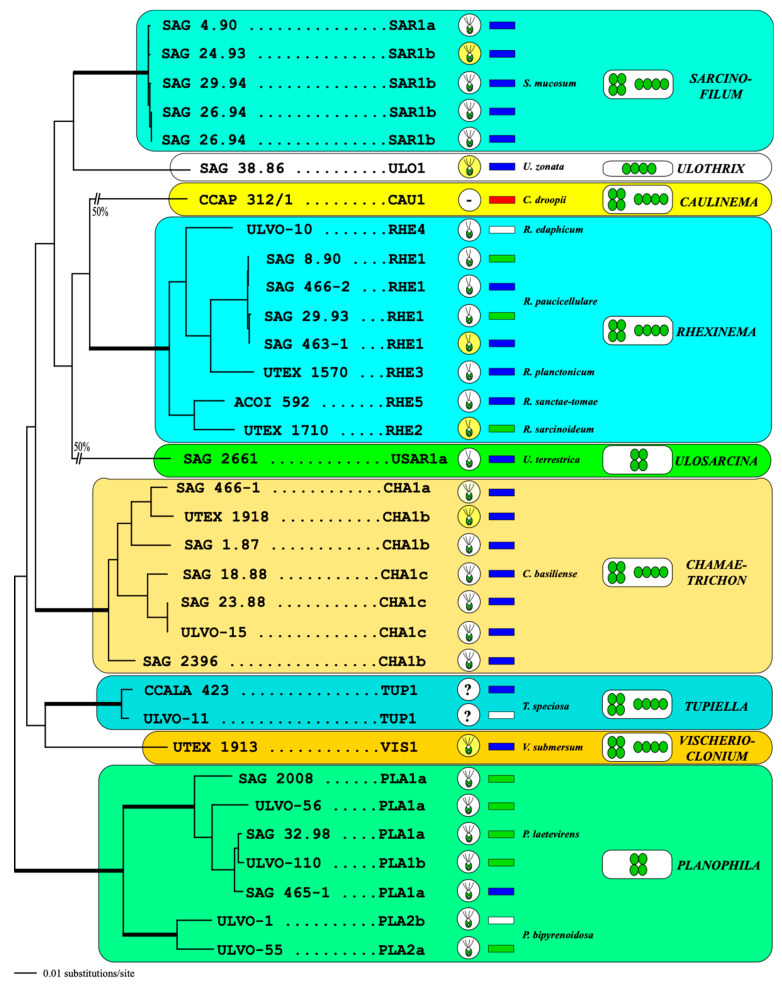
Molecular phylogeny of the Ulotrichales using a concatenated dataset of SSU, ITS, and *tuf*A. The phylogenetic tree shown was inferred using the maximum likelihood method based on the two datasets (33 taxa with 3215 bases) using PAUP 4.0a build169. For the analyses, the best model was calculated by the automated model selection tool implemented in PAUP. The settings of the best model were given as follows: GTR + I + G (base frequencies: A 0.2711; C 0.2028; G 0.2497; U 0.2764; rate matrix A-C 1.6697; A-G 3.1639; A-U 1.9207; C-G 1.2029; C-U 5.4565; G-U 1.0000) with the proportion of invariable sites (I = 0.6839) and gamma shape parameter (G = 0.6515). The branches in bold are highly supported in all analyses (Bayesian values >0.95 calculated with PHASE and MrBayes; bootstrap values >70% calculated with PAUP using maximum likelihood, neighbor joining, maximum parsimony, and RAxML using maximum likelihood). The ITS-2 barcodes are given after the strain numbers. The types of zoospores (bi- or quadriflagellated) are highlighted in circles, and the strains, which were investigated by TEM, are marked in yellow circles. The habitats are presented in squared boxes after the barcodes: freshwater (blue), terrestrial (green), marine (red), unknown (white). The morphological type of each genus is illustrated in the white boxes.

The characteristics of the nine genera belonging to the Ulotrichales are summarized in Figure 11. The polyphasic approach used in this study needs to be performed for additional lineages, such as *Ulothrix*, *Monostroma*, *Gomontia*, *Eugomontia*, *Collinsiella*, and *Gayralia*, which mostly occur in brackish and marine habitats and clearly belong to this order [50]. It is especially of great importance to clarify the phylogeny of the genus *Ulothrix*.

### 4.2. Taxonomic Revisions and Diagnoses

As demonstrated above, the newly investigated strains belong to two different genera. The strain SAG 2661 represents a new isolate of *Ulosarcina terrestrica*, which is confirmed by molecular analyses and morphological observations. The strain CCAP 312/1 represents a new genus within the order Ulotrichales, as demonstrated by the phylogenetic analyses, ITS-2 secondary structure, and morphology. As a consequence of our study, we propose the new genus *Caulinema* as follows:

***Caulinema*** gen. nov.

*Description*: Vegetative cells arranged in packages (sarcinoid) or/and as unbranched filaments, cylindrical in shape. Parietal chloroplasts containing a single pyrenoid. Uninucleate. Reproduction by vegetative cell division. *Codiolum*-like stages or akinetes present. Marine.

*Diagnosis*: Differs from other sarcinoid and filamentous genera of the Ulvophyceae by SSU and ITS rDNA and *tuf*A sequences.

*Type species* (designated here): *Caulinema droopii* sp. nov.

***Caulinema droopii*** sp. nov. (Figure 1)

*Description*: This organism has two stages: filamentous and sarcinoid with intermediate stages. Filaments consist of 20–30 vegetative cells. Filaments are very curly up to the node-like structures. Vegetative cells are cylindrical, short cylindrical, or squared, constricted at the cross walls, and sometimes with an irregular appearance. The end cells are rounded or sometimes conical. Vegetative cells possess parietal chloroplasts with a single pyrenoid surrounded by two or four large starch grains. Cell nucleus is located in the middle of the cell and badly visible. Cell wall is thin, around 0.5 µm. Every cell contains one to two large vacuoles located at the poles of the cell. Vegetative cells of this stage are 14.3–16.0 µm long × 9.2–10.5 µm wide. Reproduction is by vegetative cell division. Shortly before division, cells contain two pyrenoids. Reproduction by zoospores was not observed. *Codiolum*-like stage or akinetes are 55–60 µm × 43.7–50 µm with cell walls around 1.5–1.7 µm thick.

Package-like structures consist of two, four, or eight cells. These structures are irregularly formed and two- or three-dimensional. Eight-cell structures are from 23.6 × 23.6 up to 30.0–32.4 µm and two-celled structures are from 14.6 to 16.6 µm. Cells of packages have different shapes (conical with rounded apical sides, almost triangular, half-spherical, often trapeze-like). Cell sizes of such cells also differ from 12.9 × 14.6 µm to 6.3 × 13.3 µm.

*Diagnosis*: The genetically similar genera *Rhexinema* and *Ulosarcina* differ by the formation of longer curly filaments, larger cell size, and different ecology. *Caulinema* is a marine organism and is not able to grow on brackish or freshwater media. SSU-ITS sequences (GenBank: PQ013238) and ITS-2 Barcode: Cau1 in Figure 5.

*Note*: In our study, the strain of *Caulinema droopii* was grown on a liquid SWES medium. This alga showed no growth on agarized media or media without natural seawater.

*Holotype* (designated here): The authentic strain CCAP 312/1 is cryopreserved in a metabolically inactive state at the Culture Collection of Algae and Protozoa (CCAP), Scottish Association for Marine Science, Dunbeg by Oban, Scotland.

*Type locality*: Scotland, UK, Kames Bay, Millport, Isle of Cumbrae (55°45′18″ N, −4°54′56″ E).

*Etymology*: The species is named in honor of Dr. Michael Droop (1918–2011), who isolated this strain in 1958 and who wanted to describe this genus as *Caulinema*, a name which fits very well because of its coiled, filamentous morphology.

## Data Availability

The sequence data are available under the given accession numbers in GenBank.
